# tRNA-Derived Fragments Target the Ribosome and Function as Regulatory Non-Coding RNA in *Haloferax volcanii*

**DOI:** 10.1155/2012/260909

**Published:** 2012-12-27

**Authors:** Jennifer Gebetsberger, Marek Zywicki, Andrea Künzi, Norbert Polacek

**Affiliations:** ^1^Department of Chemistry and Biochemistry, University of Bern, Freiestraße 3, 3012 Bern, Switzerland; ^2^Graduate School for Cellular and Biomedical Sciences, University of Bern, 3012 Bern, Switzerland; ^3^Division of Genomics and RNomics, Innsbruck Biocenter, Innsbruck Medical University, Innrain 80/82, 6020 Innsbruck, Austria; ^4^Laboratory of Computational Genomics, Institute of Molecular Biology and Biotechnology, Adam Mickiewicz University, 61-712 Poznan, Poland

## Abstract

Nonprotein coding RNA (ncRNA) molecules have been recognized recently as major contributors to regulatory networks in controlling gene expression in a highly efficient manner. These RNAs either originate from their individual transcription units or are processing products from longer precursor RNAs. For example, tRNA-derived fragments (tRFs) have been identified in all domains of life and represent a growing, yet functionally poorly understood, class of ncRNA candidates. Here we present evidence that tRFs from the halophilic archaeon *Haloferax volcanii* directly bind to ribosomes. In the presented genomic screen of the ribosome-associated RNome, a 26-residue-long fragment originating from the 5′ part of valine tRNA was by far the most abundant tRF. The Val-tRF is processed in a stress-dependent manner and was found to primarily target the small ribosomal subunit *in vitro* and *in vivo*. As a consequence of ribosome binding, Val-tRF reduces protein synthesis by interfering with peptidyl transferase activity. Therefore this tRF functions as ribosome-bound small ncRNA capable of regulating gene expression in *H. volcanii* under environmental stress conditions probably by fine tuning the rate of protein production.

## 1. Introduction

Recent research revealed small nonprotein coding RNAs (ncRNAs) as pivotal players in regulatory networks shaping cellular life in all three phylogenetic domains (reviewed in [1]). Regulatory functions of ncRNAs are diverse ranging from chromosome biology, to epigenetics, transcription, and translation regulation [2]. This variety of ncRNA functions is mirrored by their complex genomics and biogenesis. Recent data suggest that a single ncRNA transcript can adopt different structures and thus perform distinct functional roles depending on different posttranscriptional processing events. Four years ago it could be shown that a functional small nucleolar RNA, which was initially processed from an mRNA intron, could function as a microRNA after further processing took place [3]. Many other reports followed expanding the list of ncRNA species that are target of further downstream processing into novel regulatory entities [4–7] thus it appears that we have just started to disentangle the hidden layers of the transcriptome.

One recent example of ncRNA processing presents the emerging group of RNA fragments derived from mature tRNAs or precursor tRNAs [8]. These tRNA-derived fragments (tRFs) have long been regarded as random byproducts of tRNA biogenesis or degradation, but are now recognized as emerging players in tRNA biology. tRFs have been uncovered by deep sequencing projects in all three domains of life (reviewed in [8]). These tRFs have been shown to be produced under specific growth conditions and differ in size and sequence, thus indicating physiological relevance. Their biological functions, however, remained largely enigmatic. Several reports show a possible involvement of tRFs in cell proliferation [9], in the siRNA and microRNA pathway [8, 10–12] and in protein biosynthesis [13–16]. In those studies presenting functional experiments, it appears that tRFs are capable of globally downregulating protein synthesis. This tRF-induced inhibition of translation is not however due to a reduced pool of genuine tRNA molecules. A recent study by Ivanov et al. suggests that specific 5′ tRFs inhibit translation initiation in oxidatively stressed human cell lines by recruiting eIF4E/G/A from capped mRNAs or eIF4G/A from uncapped mRNAs [13]. If translation initiation is the sole target for tRFs in regulating protein synthesis or whether other phases of the ribosomal elongation cycle might be targeted by this ncRNA class remains to be seen.

In this study, we present evidence that in the archaeal model species *Haloferax volcanii* tRFs are processed in a stress-dependent manner and are capable of directly binding to the ribosome. Our deep-sequencing analysis of the ribosome-associated small RNome revealed four classes of tRFs. We present experimental evidence that one of these tRFs primarily target the small ribosomal subunit and demonstrate its inhibitory role in peptide bond formation and during *in vitro* translation. This is the first report of a tRF directly binding to the key enzyme of protein synthesis and therefore tRFs in *H. volcanii* represent ribosome-targeted regulatory ncRNA species.

## 2. Material and Methods

### 2.1. Strain and Growth Conditions

*Haloferax volcanii* strain H26 was grown aerobically at 42°C in complex medium (2.9 M NaCl, 150 mM MgSO_4_ × 7 H_2_O, 60 mM KCl, 4 mM CaCl_2_, 50 mM Tris-HCl (pH 7.2), 0.45% (w/v) tryptone, 0.275% (w/v) yeast extract). Cells were subjected to different temporary and permanent stress conditions as described [17]. In the case of temporary stress *H. volcanii* precultures were grown under standard conditions to the mid exponential phase (OD_600_ = 0.5 ± 0.1) before environmental stress was applied for one respectively four hours. These stress conditions included cold shock at 30°C and heat shock at 60°C. For pH stress the cultures were either supplemented with 0.1 M Tris-HCl (pH 8.5-9.5) resulting in an elevated pH or with 12 mM Na(O)Ac for low pH conditions. Oxidative stress was induced by the addition of H_2_O_2_ to a final concentration of 0.78% (low oxidative stress) and 1.43% (high oxidative stress). For the ultraviolet (UV) stress the cells were irradiated with a UV dose of 120 J/m^2^ for 30 seconds. For osmotic stress, the cells were collected by centrifugation and resuspended in the appropriate salt stress medium (0.9 M NaCl, 1.5 M NaCl, 300 mM MgSO_4_ × 7 H_2_O). For setting a permanent stress the cells were challenged from the inoculation until an OD_600_ = 1.0 ± 0.2 was reached. These stress conditions included UV irradiation, alkaline stress (pH 8.5), and growth under different salt concentrations (1.5 M NaCl, 300 mM MgSO_4_ × 7 H_2_O) as described above. The growth was monitored at selected time points by measuring the absorbance at OD_600_. 

### 2.2. cDNA Library Generation

For the cDNA library preparation equal volumes of unstressed and stressed *H. volcanii *cells (for different stress conditions see above) were pooled and ribosomes were isolated basically as described [18] using buffer P [19] (3.4 M KCl, 100 mM Mg(OAc)_2_, 6 mM 2-mercaptoethanol, 10 mM Tris-HCl pH 7.6). Ribosome-associated RNA was size-selected (ranging from 20–500 nucleotides), extracted, precipitated with ethanol, and used for cDNA library construction as described [7]. The cDNA library was deepsequenced (max. read lengths 76 bp) using the illumina platform (FASTERIS SA).

### 2.3. Data Analysis of the Deep Sequencing Results

The analysis of the sequence reads was performed using the APART pipeline (automated pipeline for annotation of RNA transcripts) [7]. In short, the sequences were cleaned by removal of the adaptor sequences and subsequently mapped to the *H. volcanii* genome where overlapping reads were assembled into contigs. From all 73.5 Mio raw reads originally obtained only reads with a minimal length of 18 nucleotides that contained both the 5′ and 3′ adaptors were further analyzed. At the genome mapping stage only a single mismatch was allowed. 

### 2.4. Polysome Gradients

*H. volcanii* cells grown under normal conditions and permanent pH 8.5 stress condition (as described above) were collected by centrifugation, frozen in liquid nitrogen, and grounded three times in a precooled mortar. The resulting powder was resuspended in buffer A (3 M KCl, 150 mM MgCl_2_, 6 mM 2-mercaptoethanol, 10 mM Tris-HCl pH 7.6, 0.5 mM DTT, 26 *μ*M tetracycline hydrochloride) and the cell debris was removed by centrifugation (30,000 ×g, 15 min). The supernatant was treated with RNase-free DNase (10 min on ice) and subjected to a second centrifugation (30,000 ×g, 15 min). The supernatant, referred to as S30, was layered onto a linear sucrose gradient containing 10–40% (w/v) sucrose prepared in buffer A and centrifuged in a Beckman SW-41 rotor (4 h, 35,000 rpm, 4°C). Fractions containing polysomes, 50S, and 30S subunits were collected while monitoring the absorbance at 260 nm. For downstream northern blot analyses, the fractions were dialyzed two times against pure water (2 h, 4°C) and precipitated with ethanol before separation on 8% polyacrylamide gels.

### 2.5. Northern Blot Analysis

Total RNA from *H. volcanii* grown under selected conditions was isolated using TRI Reagent (Sigma Aldrich) according to the manufacturer's instruction. Size-selected ribosome-associated RNA was prepared by isolating RNA from crude ribosomes by phenol/chloroform extraction followed by size fractionation employing denaturating 8% PAGE. RNAs in the size range between 20 and 500 nt were excised from the gel, passively eluted into 0.3 M NaOAc at 4°C, and ethanol precipitated. Ribosomal particles (polysomes, 50S, or 30S subunits) were isolated using sucrose gradient centrifugation (as described above), followed by RNA extraction using phenol/chloroform and ethanol precipitation. For northern blot analysis the RNA (2.5–10 *μ*g) was separated on 8% denaturating polyacrylamide gel (7 M Urea, 1 × TBE buffer), transferred onto nylon membranes (Amersham Hybond-N^+^, GE Healthcare) using a semidry blotting apparatus (V20-SDB, Scie-Plas). After immobilizing of RNA using a UV cross-linker (BLX-254, Vilber Lourmat) the nylon membranes were prehybridized for 30 minutes in hybridization buffer (1 M sodium phosphate buffer pH 6.2, 7% SDS). DNA oligonucleotides complementary to the RNA of interest were end-labeled with [*γ*-^32^P]-ATP and T4 polynucleotide kinase. Hybridization was carried out in 1 M sodium phosphate buffer (pH 6.2, 7% SDS) over night at 52°C in a hybridization oven. Blots were washed once (room temperature) with washing buffer I (30 mM sodium citrate pH 7.0, 0.3 M NaCl, 0.1% SDS) and once (room temperature or 52°C) with washing buffer II (1.5 mM sodium citrate pH 7.0, 15 mM NaCl, 0.1% SDS) for 10 minutes each. Membranes were exposed to phosphor imaging screens. The signals were monitored with a phosphor imager (FLA-3000; Fuji Photo Film) and analyzed quantitatively with the densitometric program Aida Image Analyzer. Following DNA oligonucleotides were used for northern blotting targeting tRFs (depicted in 5′–3′ direction): Val(GAC) TCATAACCAGACTAGACCACCAACCC, Cys(GCA) CCGAACTCTGCCACCTTGGC Ser(GCT) CCAGGCTTGGCTACCGCAAC, Leu(CAG) CCAGACTTGGCTATCCCTGC, Arg(GCG) ATAGTCCACTACCCTATCAGGAC, Ala(GGC) ATCTACCCCTGATCTACGAGCCC, Leu(GAG) CCTGGCTTGGCTACCCACGC, Val(CAC) TCATAACCTGGCTAGACCACCAACCC, Gly(CCC) CTACCACTGGACCATCGGCGC, Asp(GTC) TCGTATGATGGGCCACTACACCACCCGGGC, Leu(CAA) ATTTGAACCCACGGACCCCTACGGGAGCGGAT, Ser(GGA) CCTTACCGCTCGGCCATCCTGGC, Val(GAC) TGGGTTGGGGCAGATTTGAA.

### 2.6. *In Vitro *Binding Studies

Binding studies of Val-tRF were performed using a dot blot-filtering device. For the experiments 10 pmol of *H. volcanii *ribosomal particles (70S, 50S, 30S) were incubated with 4 pmol 5′[^32^P]-end-labeled synthetic RNA (1.750 cpm/pmol) (Val-tRF 5′-GGGUUGGUGGUCUAGUCUGGUUAUGA-3′, Ile-tRF 5′. GGGCCAAUAGCUCAGUCAGGUUGAGC-3′) in 25 *μ*L binding buffer (f.c. 20 mM Hepes/KOH pH 7.6, 6 mM MgAc_2_, 150 mM NH_4_Cl, 4 mM 2-mercaptoethanol, 2 mM spermidine, 50 *μ*M spermine). As positive control *E. coli *70S were incubated with 50 *μ*g polyU-mRNA and 5′[^32^P]-end-labeled deacylated tRNA^Phe^ (7,000 cpm). After 30 min incubation on ice the reactions were filtered through a nitrocellulose membrane (0.45 *μ*m diameter) using a vacuum device, followed by two washing steps with cold binding buffer. The membrane was quantified as described above.

### 2.7. *In Vitro* Translation

For *in vitro *translation an S30 extract from *H. volcanii *was prepared as described above and stored in aliquots at −80°C. For 16 translation reactions the following components were added to 150 *μ*L S30 extract: 25 *μ*L of 10 × translation cocktail (100 mM HEPES-KOH pH 7.4, 15 mM Mg(OAc)_2_, 750 mM KOAc, 4 mM GTP, 10 mM ATP, 500 *μ*M of all amino acids except methionine and cysteine), 2.5 *μ*L creatine phosphokinase (10 mg/mL, Roche), 5 *μ*L creatine phosphate (0.6 M, Roche), 2.5 *μ*L Mg(OAc)_2_ (100 mM), and 5 *μ*L bulk brewer's yeast tRNA (10 mg/mL, Roche). Before the addition of 10 *μ*L [^35^S] cysteine/methionine (10 *μ*Ci/*μ*L), a preincubation step for 10 min at 23°C was performed. 12 *μ*L of translation mixture were used per reaction to which 50 pmol tRFs or H_2_O was added to a final volume of 15 *μ*L. *In vitro *translation was performed at 23°C for 30 min and was stopped by the addition of 1 mL 20% TCA and incubation at 95°C for 15 minutes. The TCA-precipitated proteins were filtered through glass-fiber filter and quantified by liquid scintillation counting. The background counts observed in analogous samples but in the absence of S30 extract (in average 30-fold lower than in complete *in vitro* translation reactions containing S30) was always subtracted.

### 2.8. Peptidyl Transferase Assay

Peptide bond formation activity was tested using a modified peptidyl transferase assay under fragment reaction conditions [20] with N-acetyl-[^3^H]Phe-tRNA (15,000 cpm/pmol) [21] as the donor substrate and puromycin as acceptor. The assay was performed in 25 *μ*L of high salt buffer (2 M KCl, 30 mM Tris-HCl pH 7.6, 0.4 M NH_4_Cl, 60 mM Mg(OAc)_2_, 7 mM 2-mercaptoethanol, 2 *μ*M spermidine, 0.05 mM spermine) containing 10 pmol of *H. volcanii* 70S ribosomes, respectively, 50S subunits, 0.8 pmol N-acetyl-[^3^H]Phe-tRNA, puromycin (f.c. 1 mM) and, as indicated, 100 pmol of the RNA of interest (Val-tRF, Ile-tRF, or scr 5′-GUUGUUCGCUGUGAGGUGGUGAAUUG-3′). The transpeptidation reaction was initiated by the addition of 12.5 *μ*L cold methanol (f.c. 33%) and incubated for 3.5 h on ice. In order to test for potential P-site competition between N-acetyl-[^3^H]Phe-tRNA and Val-tRF, 0.8 pmol of the peptidyl-tRNA analog was preincubated in the presence of 100 pmol Val-tRF and 33% methanol for 20 minutes on ice before the reaction was initiated by the addition of puromycin. In a control experiment, the Val-tRF was added simultaneously with the A-site substrate puromycin to ribosomal complexes that carried already a prebound N-acetyl-[^3^H]Phe-tRNA (0.8 pmol). In all cases the reaction was terminated by the addition of 5 *μ*L 10 M KOH followed by an incubation for 15 min at 37°C. After the addition of 100 *μ*L 1 M KH_2_PO_4_ the reaction product (N-acetyl-[^3^H]Phe-puromycin) was extracted into 1 mL cold ethyl acetate by vortexing for 1 min. 800 *μ*L of the upper organic phase were measured using a liquid scintillation counter.

## 3. Results

### 3.1. tRNA-Derived Fragments Are Abundant in the RNA Interactome of *H. volcanii* Ribosomes

**Figure 1:**
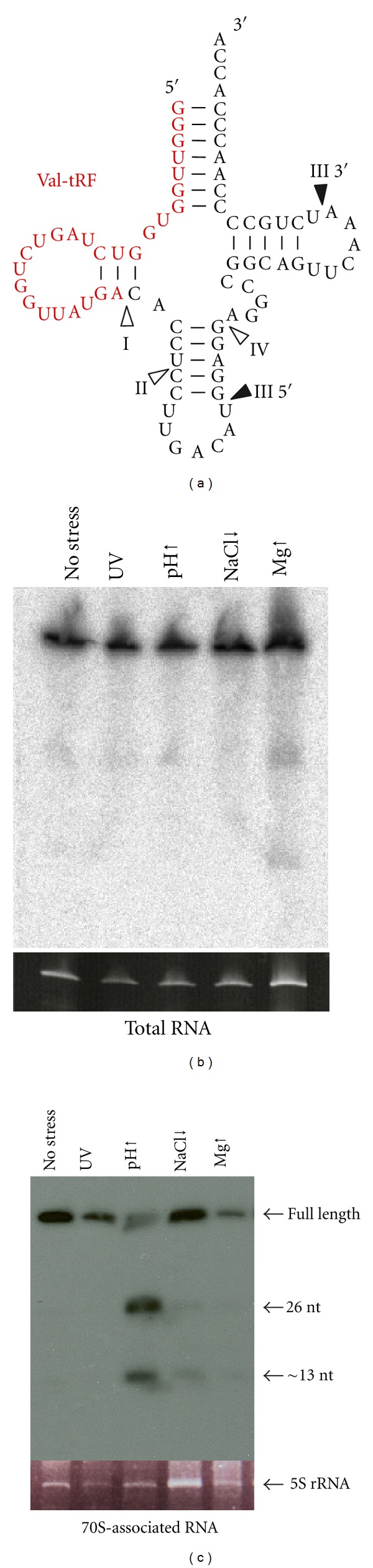
Processing and expression of Val-tRF. (a) Secondary structure of *H. volcanii* Val-tRNA with the Val-tRF depicted in red. Arrowheads indicate the processing positions for the four different observed tRF classes (I–IV). Open arrowheads on the Val-tRNA structure indicate the 3′ ends of the tRFs for class I, as well as the analogous positions for the other tRF classes II, and IV. tRFs from classes I, II, and IV are all processed from the 5′ end of mature tRNAs. Filled arrowheads mark the 5′ and 3′ ends of tRFs derived from class III. Northern blot analyses for Val-tRF were performed using (b) total RNA or (c) ribosome-associated RNA. RNA was isolated from unstressed *H. volcanii* cells (no stress), or cells grown under different permanent environmental stress conditions (ultraviolet stress (UV), high pH (pH↑), 0.9 M NaCl (NaCl↓), 300 mM MgSO_4_ (Mg↑)). Arrows indicate the full-length tRNA and the detected processing products. In all panels 5S rRNA served as internal loading control.

In the course of studying the ncRNA interactome of the archaeal ribosome, we have constructed a specialized cDNA library from small RNAs (sized 20–500 nt) that copurifies with ribosomes of *H. volcanii *under different environmental stress conditions. In order to select for functional ncRNAs putatively involved in translation regulation we have set temporary stress (UV, cold shock, heat shock, low pH, high pH, oxidative stress, low NaCl, high MgSO_4_) for one, respectively four hours, as well as permanent stress (UV, high pH, low NaCl, high MgSO_4_). For the library construction small RNAs that copurified with *H. volcanii *ribosomes isolated from stressed and unstressed cultures were reverse transcribed into cDNA. The subsequent deep sequencing yielded 73.5 million raw reads which were analyzed using the APART pipeline [7], ending up with 19.2 million reads for downstream analysis which were grouped into 6.250 putative ribosome-associated ncRNA candidates (our unpublished data). Among other potential novel ncRNAs we significantly observed the emersed presence of tRNA-derived fragments (tRFs) originating from 14 different tRNAs (Table 1). Based on their sizes and processing ends we have categorized them into four different classes of which class I, with a size distribution of 20–26 nt and the 3′-end located in the D-stem, is the most prominent one (1.3 million reads). Interestingly, tRFs of all classes (with the exception of class IV) are processed from the 5′ end of the mature tRNA (for a schematic representation see Figure 1(a)). No reads encompassing 3′-derived tRFs were obtained in our library. The presence of tRFs in a library constructed from the ribosome-bound RNome suggests that 5′ tRFs (but not 3′ tRFs) are capable of interacting with the translation machinery. Bioinformatic analyses failed to detect any correlations between the processed tRNAs and the codon usage statistics in *H. volcanii*.

### 3.2. Valine tRF Is Highly Abundant and Associates with the Ribosome

With more than 1.1 million reads (85% of all detected tRF reads) a 26-residue-long fragment deriving from valine tRNA showed unexpected high abundancy (Table 1). Whereas in *H. volcanii *four genes give rise to Val-tRNAs, our observed tRF originates from two paralogous tRNA genes decoding for valine (GAC), positioned adjacent in the genome. To confirm the presence of this tRF and to investigate its potential association with ribosomes, northern blot analyses were performed. While only very faint bands for Val-tRNA processing products were seen when total RNA was used for northern blotting (Figure 1(b)), clear bands of the expected size of about 26 nucleotides were detected in blots using ribosome-associated RNAs (Figure 1(c)). Obviously the Val-tRF is enriched in the latter RNA preparation thus supporting our assumption of Val-tRF being a ribosome-bound RNA species. Even extended incubation times with a northern blot probe targeting the 3′ part of Val-tRNA failed to identify any processing product (data not shown). Thus only the 5′ tRF has the potential to bind to ribosomes. From Figure 1(c), it is also obvious that Val-tRF is processed differently in response to diverse stress conditions. At elevated pH this tRF is most abundant. 

### 3.3. tRFs Are Differentially Expressed under Stress

**Figure 2:**
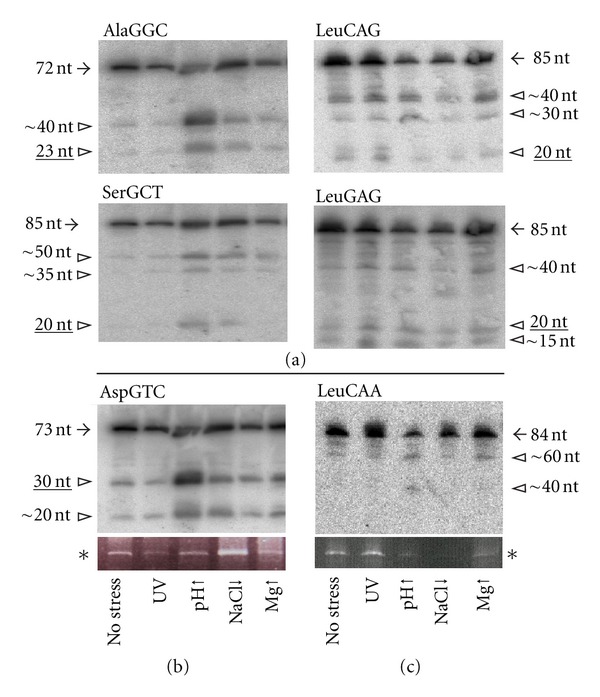
tRFs are present in the ribosome-associated RNome. Northern blot analyses of tRFs confirm the presence of class I (a), class II (b), and class III (c) tRFs in the ribosome-associated RNA fraction. The full-length tRNA signals are depicted by black arrows and tRFs by open arrow heads. Approximate lengths of the fragments, as deduced from RNA markers, are indicated. tRFs corresponding to the sequence reads of the cDNA library (Table 1) are underlined. RNA was isolated from unstressed *H. volcanii* cells (no stress), or cells grown under different permanent environmental stress conditions (ultraviolet stress (UV), high pH (pH↑), 0.9 M NaCl (NaCl↓), 300 mM MgSO_4_ (Mg↑)). The 5S rRNA (asterisk) of ethidium bromide stained gels served as loading controls.

In order to clarify whether tRFs from all four classes found in our library are part of the ribosome-derived RNome, northern blot analyses were performed (Figure 2). It turned out that indeed signals for almost all tested tRFs were observed; however, the processing pattern as well as the abundance was distinct. While the class I tRF derived from Ala-tRNA showed a very similar processing pattern to Val-tRF at elevated pH as the condition of most significant tRF processing, the class I tRFs from Leu-tRNA were markedly different. Nevertheless, the fact that these tRFs could be readily detected via northern blot analyses on ribosome-derived RNA suggests that the tRFs identified in our genomic screen are capable of interacting with the ribosome in *H. volcanii*.

### 3.4. Valine tRF Primarily Binds to the Small Ribosomal Subunit

**Figure 3:**
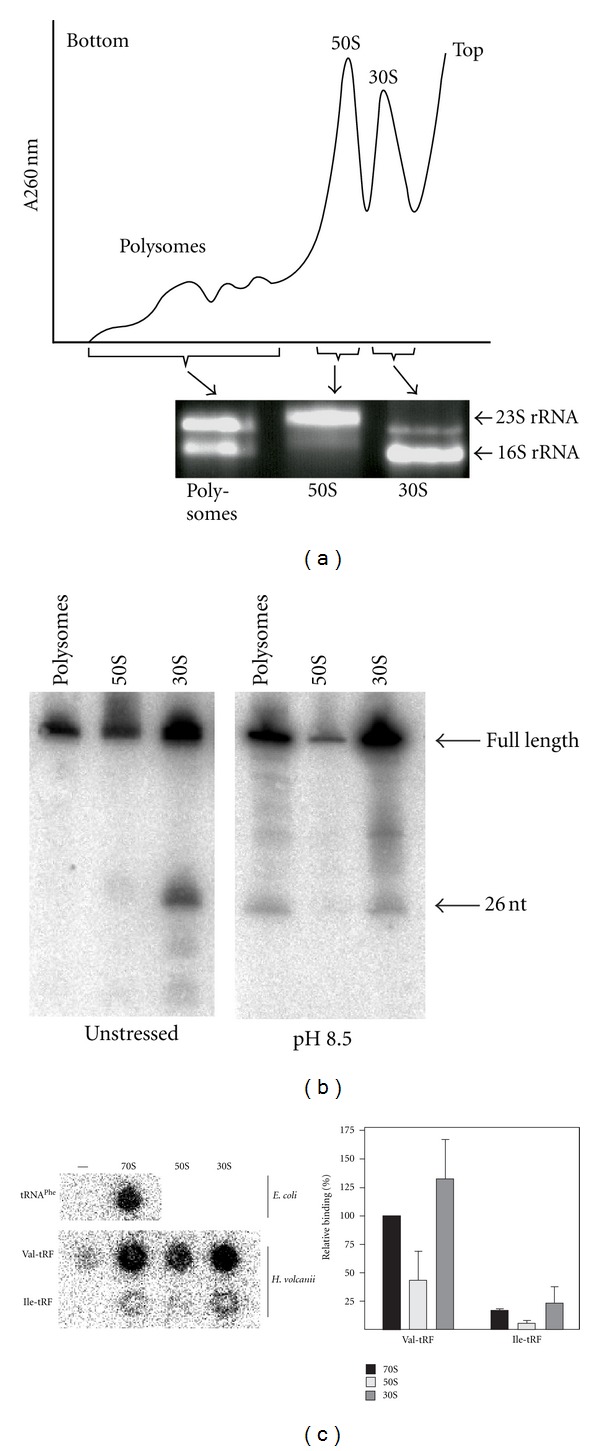
Val-tRF associates with ribosomes *in vitro *and *in vivo.* (a) A representative polysome gradient of *H. volcanii.* Fractions containing polysomes, 50S, or 30S subunits were collected and used for northern blot analyses. The identity of the individual fractions was confirmed by agarose gel electrophoresis. (b) The presence of the Val-tRF in the different gradient fractions was investigated by northern blot analysis using RNA from unstressed cells or from cultures incubated at high pH (pH 8.5). Arrows indicate the full-length Val-tRNA and the 26 nt long fragment detected in the cDNA library. (c) *In vitro *filter binding studies of radiolabelled synthetic Val-tRF on ribosomal particles (70S, 50S, 30S) from *H. volcanii* (left panel). As a positive control, binding of tRNA^Phe^ to *E. coli *70S was monitored. To confirm specific binding of Val-tRF an equally long fragment of isoleucine tRNA (Ile-tRF), an RNA sequence not found in our cDNA screen, served as negative control. (Right panel) Quantification of relative binding whereas association of Val-tRF to 70S was normalized to 100%. Signals measured in the absence of any ribosomal particles (-) were subtracted from all experimental points. Error bars show the mean and standard deviation of at least four independent experiments.

To more precisely investigate the tRF interaction with the ribosome, polysome gradient analyses and* in vitro* binding studies were performed. *H. volcanii* cell lysates were passed through a linear sucrose gradient in order to separate the polysomal fraction from nontranslating 70S ribosomes and ribosomal subunits. RNA isolated from these fractions was used for northern blot analysis employing a radiolabelled antisense probe targeting Val-tRF (Figures 3(a) and 3(b)). The data revealed that Val-tRF was primarily comigrating with the 30S subunit fraction (Figure 3(b); unstressed and pH 8.5 stress) in density gradients. Only after high pH stress, Val-tRF signals were also obtained in the polysomal fraction (Figure 3(b)). This is evidence for a putative functional role of Val-tRF during stress response. To more directly investigate ribosome-association, radiolabelled synthetic Val-tRF was bound to purified *H. volcanii* ribosomes or subunits in a filter binding setup. The binding data show that Val-tRF can indeed associate with 70S ribosomes and 30S subunits, while 50S particles were less efficiently targeted (Figure 3(c)). Obviously, no additional cellular proteins are required for ribosome binding. As a negative control, an analogous tRF deriving from a comparable region of isoleucine tRNA, a fragment that was not represented in our deep-sequence library, was used. This isoleucine tRNA-derived fragment (Ile-tRF) was unable to bind to the ribosome in a comparable manner (Figure 3) thus highlighting binding specificity of the Val-tRF.

### 3.5. Valine tRF Inhibits *In Vitro* Translation by Interfering with Peptide Bond Formation

**Figure 4:**
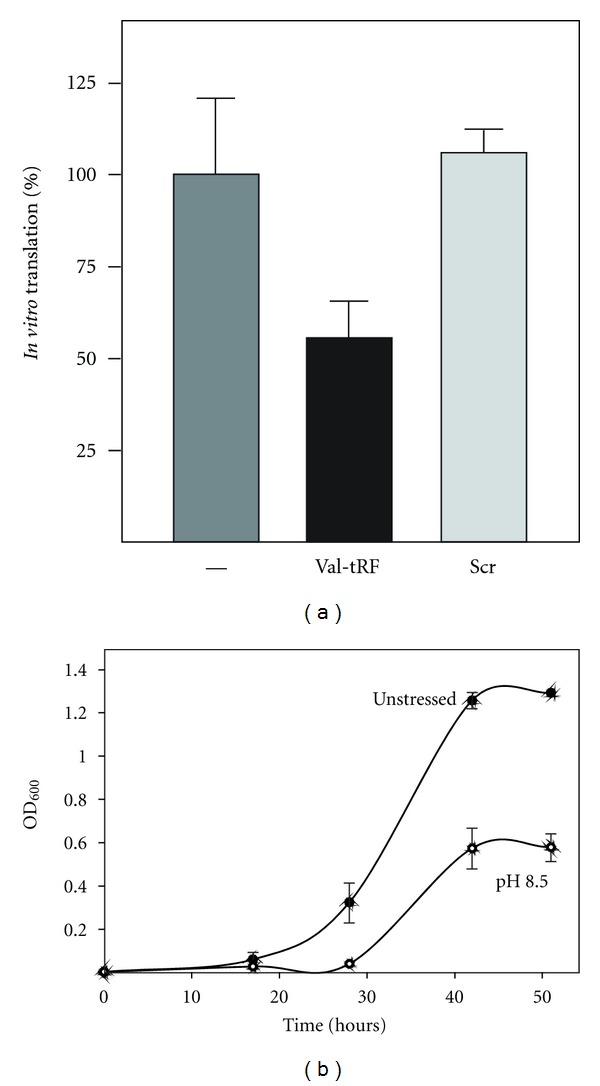
Effects of Val-tRF or elevated pH on protein synthesis and cell growth. (a) Val-tRF inhibits *H. volcanii in vitro *translation. The relative amount of radiolabeled proteins in the absence (-) or in the presence of 3.3 *μ*M Val-tRF is shown. The scrambled version of the Val-tRF (scr) served as specificity control. In all cases, the background values measured in reactions without S30 extracts were subtracted from all experimental points. Error bars represent the mean and standard deviation of at least three independent experiments. (b) Cell growth of unstressed *H. volcanii* cultures and of cells grown under alkaline conditions (pH 8.5) is shown. Cell density was measured at 600 nm and the average values of two independent growth curves each and their standard deviations are given.

The observation that Val-tRF strongly binds to ribosomes led to the speculation of a potential effect as regulatory ncRNA during protein biosynthesis. To clarify this we set up an *in vitro* translation system for *H. volcanii *and quantified the amount of synthesized proteins in the presence or absence of Val-tRF. Repeatedly, Val-tRF decreased translation efficiency of the total *H. volcanii* mRNA population by about 45% (Figure 4(a)). Importantly a 26-mer composed of a scrambled sequence of the Val-tRF did not show any inhibition of protein synthesis, thus hinting at a sequence-specific effect. This effect of Val-tRF on *in vitro* translation also helps explaining the observed severe growth defect, when *H. volcanii *was grown in alkaline media (Figure 4(b)). Under these environmental conditions Val-tRF is most strongly associated with ribosomes *in vivo* (Figure 1(b)).

**Figure 5:**
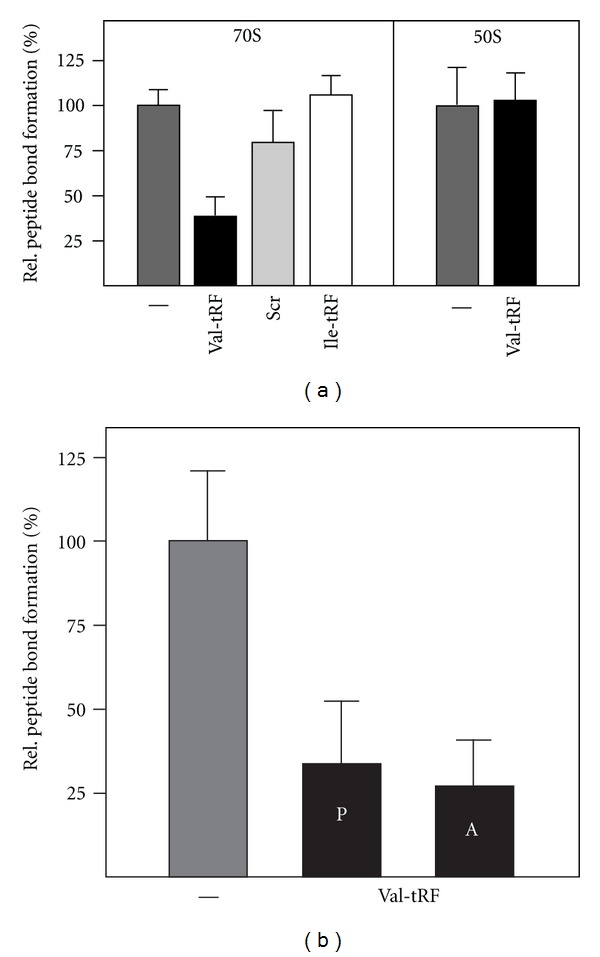
Val-tRF inhibits peptide bond formation. (a) Peptidyl transferase reactions catalyzed by *H. volcanii* 70S ribosomes (left panel) or 50S subunits (right panel) in the absence (-) or presence of 2.7 *μ*M Val-tRF (a 10-fold molar excess) were performed as described in Material and Methods. The scrambled version of the Val-tRF (scr) as well as Ile-tRF served as specificity controls. The relative amount of the reaction product N-acetyl-[^3^H]Phe-puromycin (in %) is shown. The product formed in the absence of any tRF (-) was taken as 100%. (b) Val-tRF inhibits peptide bond formation to the same extent regardless if it was added simultaneously with the P-site donor substrate (P) or with the A-site acceptor substrate (A). In all cases, the background values measured in reactions without any ribosomal particles were subtracted from all experimental points. Error bars represent the mean and standard deviation of three to five independent experiments.

Protein synthesis is a multistep process involving the orchestrated action of several protein cofactors, including initiation, elongation, and termination factors. To reveal whether the ribosome itself or any of the translation factors needed for protein synthesis are inhibited by Val-tRF, we performed a peptidyl transfer assay utilizing purified *H. volcanii* ribosomes. The transpeptidation assay was performed using N-acetyl-[^3^H]Phe-tRNA as donor and the antibiotic puromycin as acceptor substrate. In this experimental setup, Val-tRF inhibited the extent of peptide bond formation by about 60%, while the scrambled sequence of the Val-tRF had hardly any inhibitory effect (Figure 5). As second specificity control served the Ile-tRF previously used in the binding studies (see Figure 3(c)). As shown before this RNA was unable to efficiently bind to *H. volcanii* ribosomes (Figure 3(c)) and had also no inhibitory effects on the peptidyl transferase reaction (Figure 5). 

To functionally define the site of action of the Val-tRF, the puromycin reaction was repeated under identical reaction conditions, but in the presence of 50S subunits alone. In this experimental setup, Val-tRF did not inhibit transpeptidation, thus confirming that the prime binding target for Val-tRF is the small ribosomal subunit. A possible mechanism for inhibiting the puromycin reaction is that the Val-tRF competes with the P-site substrate N-acetyl-[^3^H]Phe-tRNA for ribosome binding. This could occur if the Val-tRF would be able to refold into a hairpin structure resembling an anticodon stem-loop. Even though secondary structure analyses by M-fold did not indicate any stable stem-loop structure of Val-tRF (data not shown), we decided to experimentally test the P-site competition hypothesis. Therefore we slightly modified the peptidyl transferase assay namely; we incubated N-acetyl-[^3^H]Phe-tRNA simultaneously with a 125-fold excess of Val-tRF for 20 minutes before the reaction was initiated by the addition of puromycin. Under these conditions significant inhibition of transpeptidation was observed (Figure 5(b)), thus potentially P-site competition occurred. If this Val-tRF triggered inhibition was indeed the cause of a P-site competition, we expected no reduction in the peptidyl transferase activity when Val-tRF was added together with the A-site substrate puromycin to a ribosomal complex already prebound with N-acetyl-[^3^H]Phe-tRNA at the P-site. However, also under these experimental conditions a significant drop of peptide bond formation was observed (Figure 5(b)). Since the puromycin assay under these conditions has been shown before to be a single-turnover reaction [22], we conclude that Val-tRF does not interfere with peptide bond formation due to a competition with N-acetyl-[^3^H]Phe-tRNA for the ribosomal P-site.

## 4. Discussion

Translation is essentially the last step where regulatory input can be given in the course of gene expression. Typically translation initiation, the rate limiting step of protein biosynthesis, is targeted by regulatory molecules which lead to either global or mRNA-specific effects on protein production [23]. Many regulatory proteins have been identified whose actions result in fine tuning protein synthesis rates thus enabling organisms and cells to adapt to changing environments or to fulfill tissue specific tasks. More recently also ncRNAs have been added to the list of regulatory entities that modulate translation with microRNAs and antisense RNAs being the most prominent classes in eukaryal and prokaryotic species, respectively [24]. Interestingly essentially all of these ncRNAs that control protein biosynthesis target the mRNAs rather than the ribosome itself, with 7SL RNA and tmRNA as notable exceptions (reviewed in [25, 26]). This is unexpected given the central position the ribosome, a highly conserved multifunctional ribonucleoprotein (RNP) complex plays during gene expression. The fact that more than 50% of all natural antibiotic target the ribosome clearly shows that this highly conserved RNA enzyme is in principal an ideal target for manipulating the rate of gene expression [27]. Furthermore, it is assumed that the ribosome is an evolutionary relic from the RNA world where regulatory input likely was given by nonproteinous molecules, such as small ncRNAs. 

In order to investigate whether or not small ncRNAs are still used by contemporary cells and organisms to regulate protein production, we have analyzed the RNA interactome of translating ribosomes. Our previous genomic screen in *S. cerevisiae *already indicated that such RNAs do exist [7]; thus we set out to look for this potentially novel class of ncRNAs in the halophilic archaeon *Haloferax volcanii*. Ribosomes were isolated from environmentally stressed cells and the small RNAs that copurified were analyzed by deep-sequencing analysis. Besides numerous other potential ncRNA regulators found to be associated with the ribosomes (our unpublished data), 26% of all obtained reads originated from tRNA loci. This by itself was not unexpected since tRNAs are abundant ncRNAs and well-known substrates of the ribosomes. More unforeseen, however, was the detection of 1.3 million reads originating from tRNA fragments (tRFs) (Table 1). 14 different tRNA species were found to be processed into tRFs almost exclusively from their 5′ ends (Figure 1). Stress-depandant processing of tRFs has been reported before in other model systems including yeast [7, 28], human cell lines [13, 15, 29], pumpkin [16], *Aspergillus* [14], *H. volcanii* [30], and others (see [8] and references therein). While the enzymes responsible for such tRNA processing in yeast (Rny1) [31] and mammalian cells (angiogenin) [29] have just very recently been identified, essentially nothing is known about tRF processing in archaea. In most of the cases the cellular target and the molecular function of tRFs remained unclear. In this paper, we show that tRFs from *H. volcanii* bind to the ribosome *in vivo* and *in vitro* (Figures 1–3). Importantly only tRFs derived from the 5′-part of genuine tRNA were capable for this interaction. With 1.1 million essentially identical sequence reads, the 26 nucleotide long tRF from the Val-tRNA (GAC) locus was by far the most abundant tRF found in our screen (Table 1). The northern blot signal for Val-tRF was faint when total cellular RNA was used (Figure 1(b)) but increased significantly when ribosome-associated RNA was employed, especially upon alkaline stress (Figure 1(c)). These findings suggest that tRNA processing is stress-independent, a finding that is in good agreement with a very recent study by Saikia et al. [32] and previous reports [5, 8, 9, 14, 16] and shows that Val-tRF is preferentially bound to ribosomes upon shifting the cells to elevated pH. Additional enrichment for functional ribosomal particles by polysome gradient analysis led to a further increase of the northern blot signal for Val-tRF (Figure 3(b)). This polysome profiling approach and direct binding studies demonstrated the association of this tRF primarily with the small ribosomal subunit (Figure 3). Only under elevated pH conditions, Val-tRF signals were also found in the polysome fraction. Val-tRF was able to inhibit the translation of *H. volcanii* proteins *in vitro* (Figure 4(a)), most likely by interfering with peptide bond formation (Figure 5). Inhibition of amide bond synthesis was only seen in the context of 70S ribosomes, but not in 50S subunits (Figure 5). These functional data support our findings that Val-tRF mainly binds to and acts on the small ribosomal subunit of *H. volcanii*. Since Val-tRF shifts into the polysomal fraction during pH stress (Figure 3(a)), it is possible that this interaction downregulates translational activity by interfering with efficient transpeptidation. This possible functional scenario can explain the slow growth phenotype of *H. volcanii* at elevated pH (Figure 4(b)), conditions where Val-tRF most strongly associates with ribosomes (Figure 1(c)).

The ever growing class of tRFs is not by far a homogenous group of RNA molecules. Markedly different processing patterns, sequence motifs, stress response behaviors, and (putative) mode of actions have been reported. In human cells, it was shown that a run of consecutive G residues on the 5′-end of tRFs was crucial for inhibiting the translation initiation step [13]. However, most other tRF identified so far lack any 5′ homopolymeric stretches of nucleotides. Also the fate of the two potential tRNA halves (5′ or 3′) after processing seems to be diverse. In some cases both tRNA halves are stable in the cell and thus can be considered ncRNA candidates [7, 14, 33, 34] while in other cases ([7, 13] and this study), primarily 5′ tRNA fragments were observed. Even within one species, the pattern of tRF biogenesis appears to vary evidently. A recent study by the Marchfelder group reported on the detection of 11 different tRFs in *H. volcanii *[30]. In that study total RNA of differently grown *H. volcanii* cells was used to generate the cDNA library for deep-sequencing analysis. Significantly, not a single tRF was identical when compared to our data despite the fact that the same archaeal strain was used and similar stress conditions were applied. Furthermore the tRF processing pattern in Heyer et al. was strikingly different [30]. All but one tRF derived from the 3′-trailer sequence of pre-tRNAs, and only one from the 5′-part of the tRNA. In our screen, however, nearly all tRFs were processed out from the 5′-ends of full length tRNA. One likely explanation for these apparent differences is that the ribosome selects specifically only tRFs originating from the 5′-ends of tRNAs. This strongly indicates that markedly different RNomes have been analyzed in these two studies, possibly reflecting the highly diverse ncRNA biology of tRNA-derived fragments. Additionally, recent evidence demonstrated [35] that the fraction of biologically functional ncRNA molecules can be increased when cDNA libraries were prepared from RNP particles (our approach) compared to naked total RNA [30]. 

In summary, we describe herein one of the first direct cellular target for tRFs, namely the ribosome. Furthermore we present evidence that the 26-residue-long Val-tRF is processed in a stress-dependent manner, primarily at elevated pH values and show that ribosome association of this RNA species downregulates protein biosynthesis. Thus we think that this RNA species fulfills all criteria to be referred to as regulatory ncRNA in *H. volcanii*.

## Figures and Tables

**Table 1:** Overview on tRF sequence reads in the *H. volcanii* cDNA library.

			tRNA-derived fragment					
	tRNA	tRNA #	Begin	End	Sequence (5′–3′)	Length	Reads	Reads/million
Class I	ValGAC	19	2,328,216	2,328,241	GGGUUGGUGGUCUAGUCUGGUUAUGA	26 nt	1,149,000	59,548
	ValGAC	20	2,328,336	2,328,361				
	CysGCA	13	1,603,383	1,603,402	GCCAAGGUGGCAGAGUUCGG	20 nt	95,214	4,935
	SerGCT	29	2,620,371	2,620,352	GUUGCGGUAGCCAAGCCUGG	20 nt	44,220	2,292
	LeuCAG	30	2,617,916	2,617,897	GCAGGGAUAGCCAAGUCUGG	20 nt	42,006	2,177
	ArgGCG	43	452,423	452,401	GUCCUGAUAGGGUAGUGGACUAU	23 nt	5,144	267
	AlaGGC	10	1,048,559	1,048,581	GGGCUCGUAGAUCAGGGGUAGAU	23 nt	4,332	225
	LeuGAG	31	2,564,858	2,564,839	GCGUGGGUAGCCAAGCCAGG	20 nt	3,606	187
	ValCAC	33	2,264,075	2,264,050	GGGUUGGUGGUCUAGCCAGGUUAUGA	26 nt	3,576	185
	GlyCCC	35	1,733,386	1,733,366	GCGCCGAUGGUCCAGUGGUAG	21 nt	91	5

Class II	AspGTC	48	311,827	311,755	GCCCGGGUGGUGUAGUGGCCCAUCAUACGA	30 nt	2,094	109
	AspGTC	49	311,725	311,696		30 nt	1,962	102

Class III	LeuCAA	26	2,693,522	2,693,553	AUCCGCUCCCGUAGGGGUCCGUGGGUUCAAAU	32 nt	1,425	74

Class IV	SerGGA	47	408,423	408,380	GCCAGGAUGGCCGAGCGGUAAGGCGCACGCCUGGAAAGCGUGUU	44 nt	291	15

The tRNA genes for which tRFs have been detected are shown. Columns “begin” and “end” list the position of the respective tRF on the *H. volcanii* chromosome. Sequence, length, and read numbers for each tRF are depicted. The presence of all tRFs could be verified by northern blot analyses (Figure 2 and data not shown), with the exception of ValCAC, GlyCCC, and SerGGA.
